# Supplementary material for: The Interaction of Cyclic Naphthalene Diimide with G-Quadruplex under Molecular Crowding Condition
Source: Molecules. 2020 Feb 4;25(3):668. doi: 10.3390/molecules25030668 (PMC7037305; doi:10.3390/molecules25030668)
Supplement: Supplementary file 1 [file molecules-25-00668-s001.pdf]

Electronic Supplementary Information

The interaction of cyclic naphthalene diimide with G-quadruplex under molecular crowding condition

Tingting Zou[1,2], Shinobu Sato[1,2], Rui Yasukawa[1], Ryusuke Takeuchi[1], Shunsuke Ozaki[1], Satoshi Fujii[3], Shigeori Takenaka[1,2,*]

[1] Department of Applied Chemistry, Kyushu Institute of Technology, Fukuoka, 804-8550, Japan
[2] Research Center for Bio-microsensing Technology, Kyushu Institute of Technology, Fukuoka, 804-8550, Japan
[3] Department of Bioscience and Bioinformatics, Kyushu Institute of Technology, Fukuoka 820-8502, Japan
* Correspondence: shige@che.kyutech.ac.jp; Tel.: +81-93-884-3322

Table of contents

P2	**Figure S1.** (a) HPLC and (b) MALDI-TOF-MS confirmation of **2**<br>**Figure S2.** ^{1}H-NMR confirmation of **2**
P3	**Figure S3.** ^{13}C-NMR confirmation of **2**<br>**Figure S4.** (a) HPLC and (b) MALDI-TOF-MS confirmation of **3**
P4	**Figure S5.** ^{1}H-NMR confirmation of **3**<br>**Figure S6.** ^{13}C-NMR confirmation of **3**
P5	**Figure S7.** CD spectra of **1** and **3** recognizing telomere G1 and c-*myc* under dilute condition and molecular crowding condition
P6	**Figure S8.** ITC fitting curve of Telomere G1 and c-*myc* with **1**, **2** or **3** under dilute condition.<br>**Figure S9.** ITC fitting curve of Telomere G1 and c-*myc* with **1**, **2** or **3** under molecular crowding condition.
P7	**Figure S10.** UV-Vis absorbance spectra of Telomere G1 and c-*myc* with **1**, **2** or **3** under dilute condition.
P8	**Figure S11.** UV-Vis absorbance spectra of Telomere G1 and c-*myc* with **1**, **2** or **3** under molecular crowding condition.
P9	**Figure S12.** cNDI derivatives enhance the formation of parallel c-*myc* G-quadruplex under cation-deficient molecular crowding condition.
P10	**Table S1.** CD spectra of Telomere G1 under different condition.

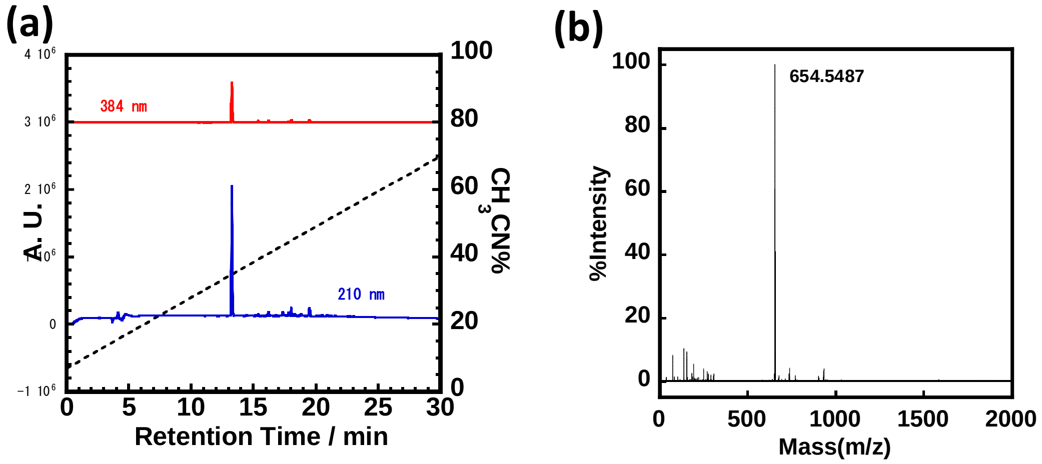

Figure S1. (a) HPLC and (b) MALDI-TOF-MS confirmation of **2.** HPLC conditions; The concentration of acetonitrile (CH_3CN) was changed (from 7% to 70%, 30 min) in water containing 0.1% trifluoroacetic acid at 40 °C.

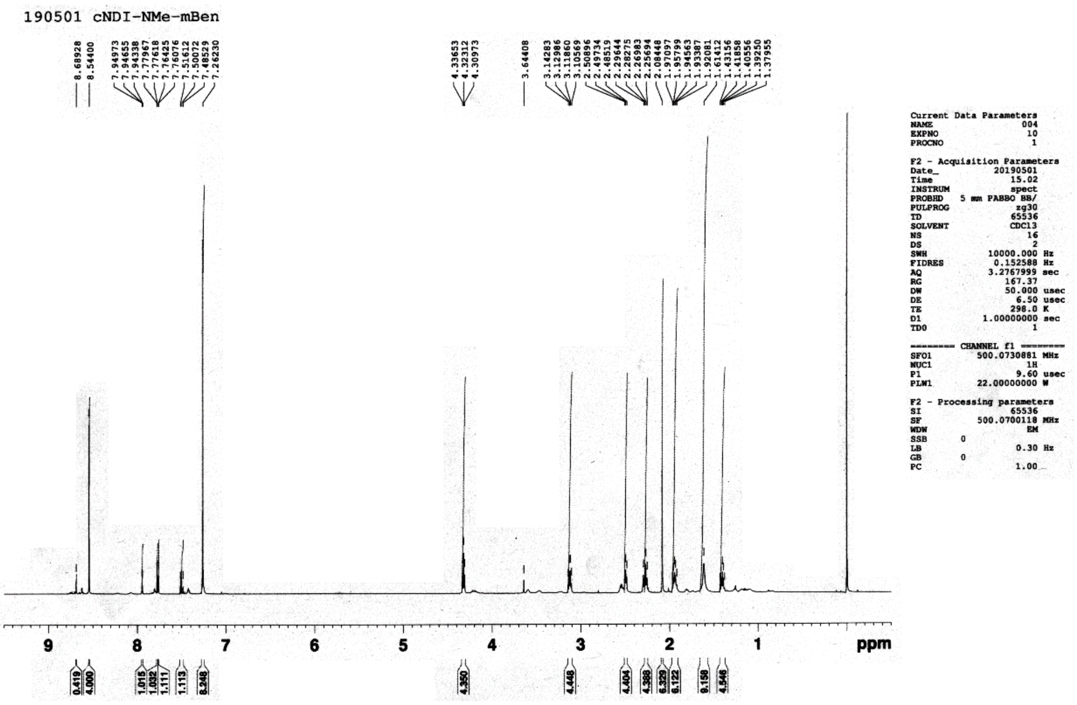

Figure S2. ^{1}H-NMR confirmation of **2**.

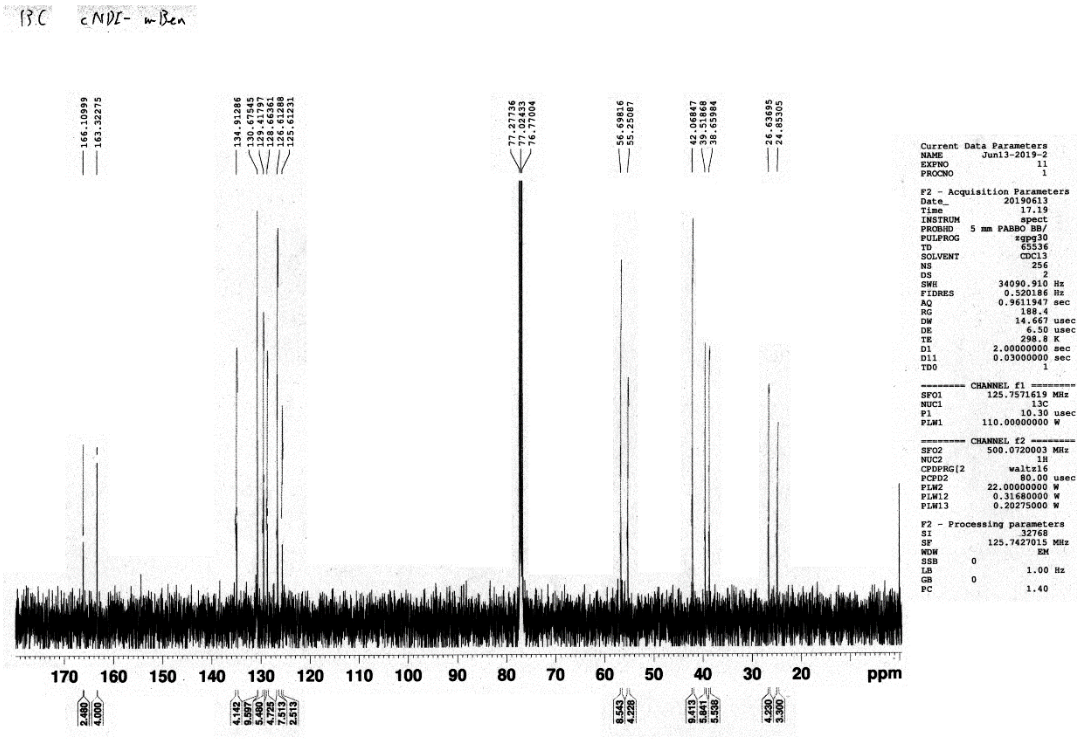

Figure S3. ^{13}C-NMR confirmation of **2**.

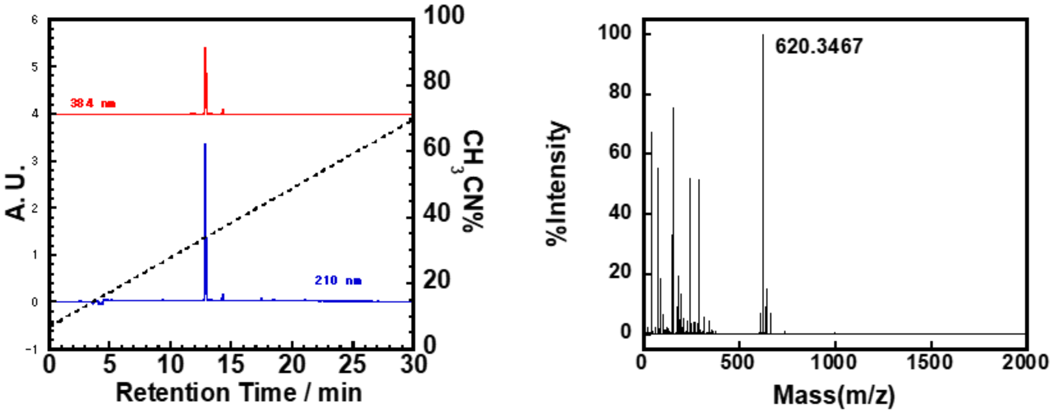

Figure S4. (a) HPLC and (b) MALDI-TOF-MS confirmation of **3**. HPLC conditions; The concentration of acetonitrile (CH_3CN) was changed (from 7% to 70%, 30 min) in water containing 0.1% trifluoroacetic acid at 40 °C.

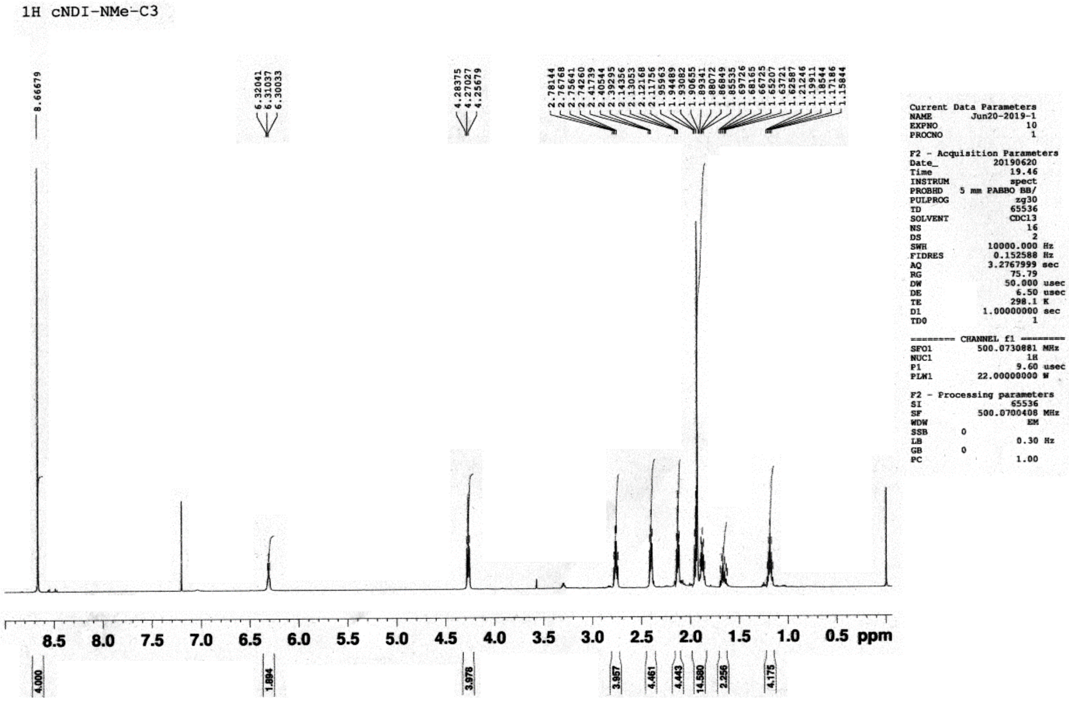

Figure S5. ^{1}H-NMR confirmation of **3**.

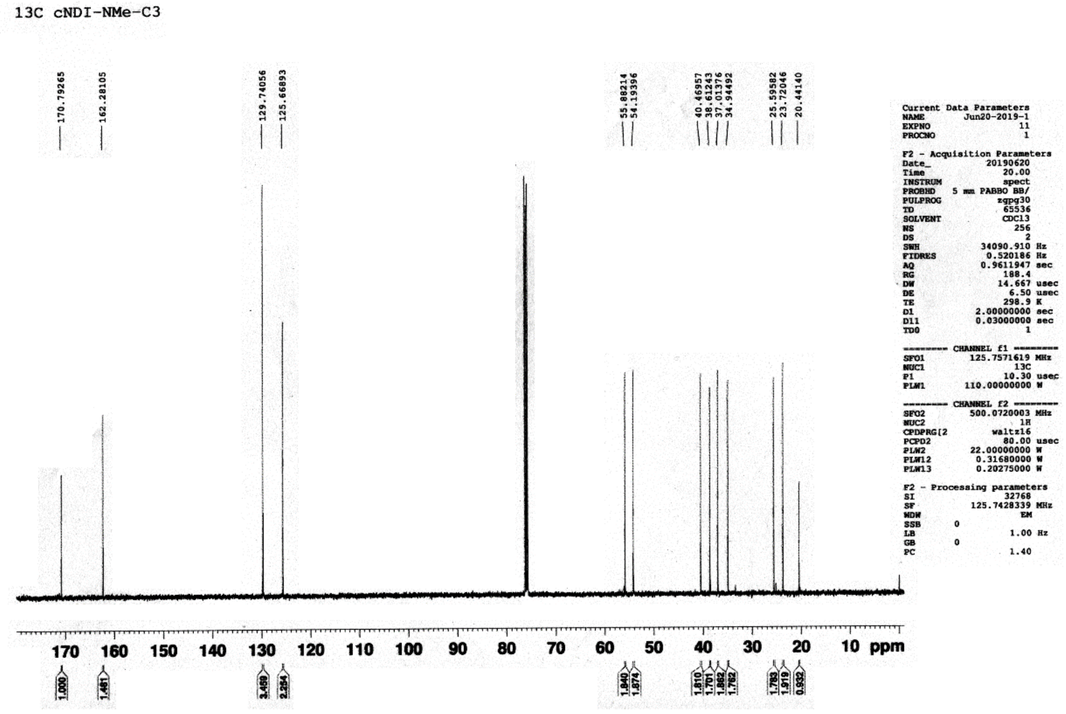

Figure S6. ^{13}C-NMR confirmation of **3**.

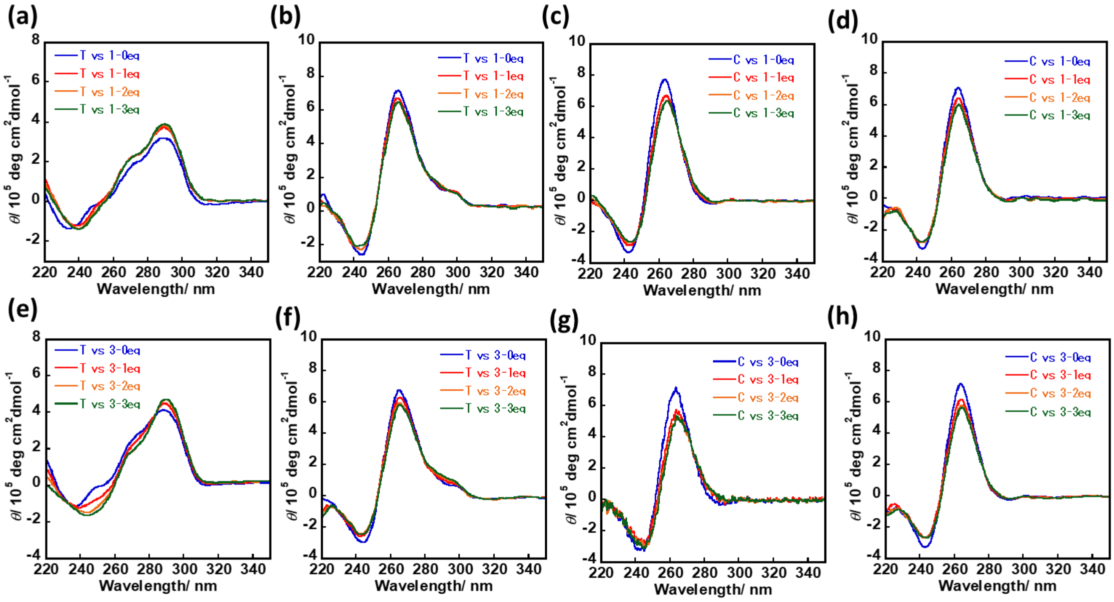

Figure S7. CD spectra of **1** recognizing telomere G1 (a) and c-*myc* (c) under dilute condition, and for telomere G1 (b) and c-*myc* (d) under molecular crowding condition; CD spectra of **3** recognizing telomere G1 (e) and c-*myc* (g) under dilute condition, and for telomere G1 (f) and c-*myc* (h) under molecular crowding condition. Dilute condition: 50 mM Tris-HCl buffer (pH 7.4) and 100 mM KCl; Molecular crowding condition: 50 mM Tris-HCl buffer (pH 7.4), 100 mM KCl, and 40%(v/v) PEG 200. T: telomere G1; C: c-*myc*; eq: equivalent.

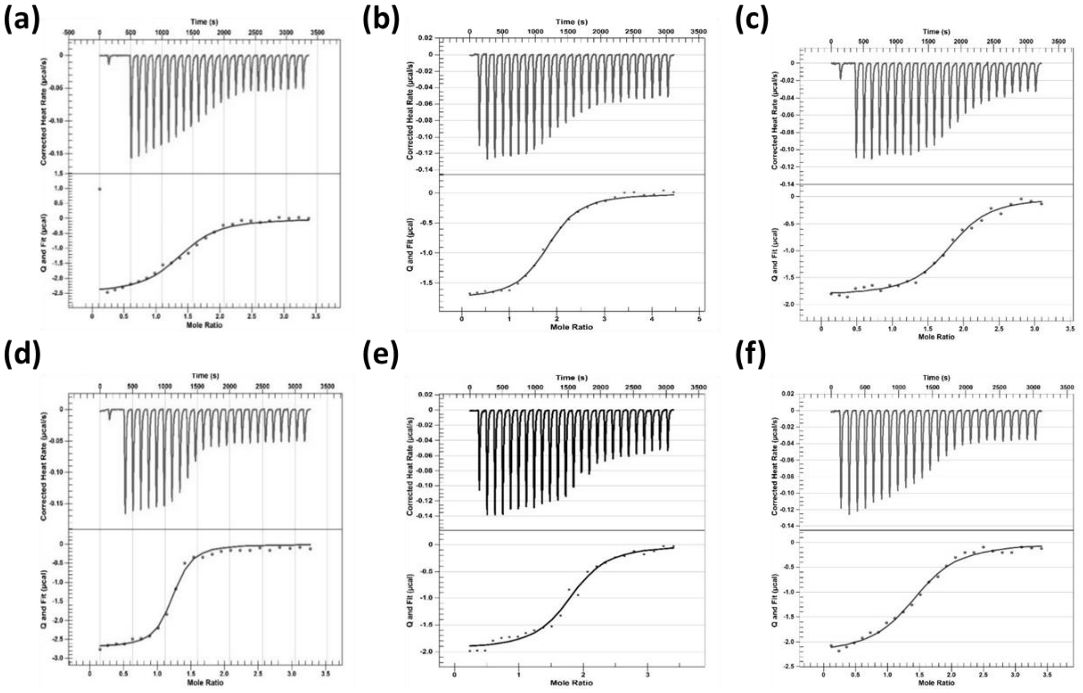

Figure S8. ITC fitting curve of Telomere G1 with **1** (a), **2** (b) and **3** (c), c-*myc* with **1** (d), **2** (e) and **3** (f) under dilute condition. 50 mM KH_2PO_4-K_2HPO_4 buffer (pH 7.0) at 25 °C

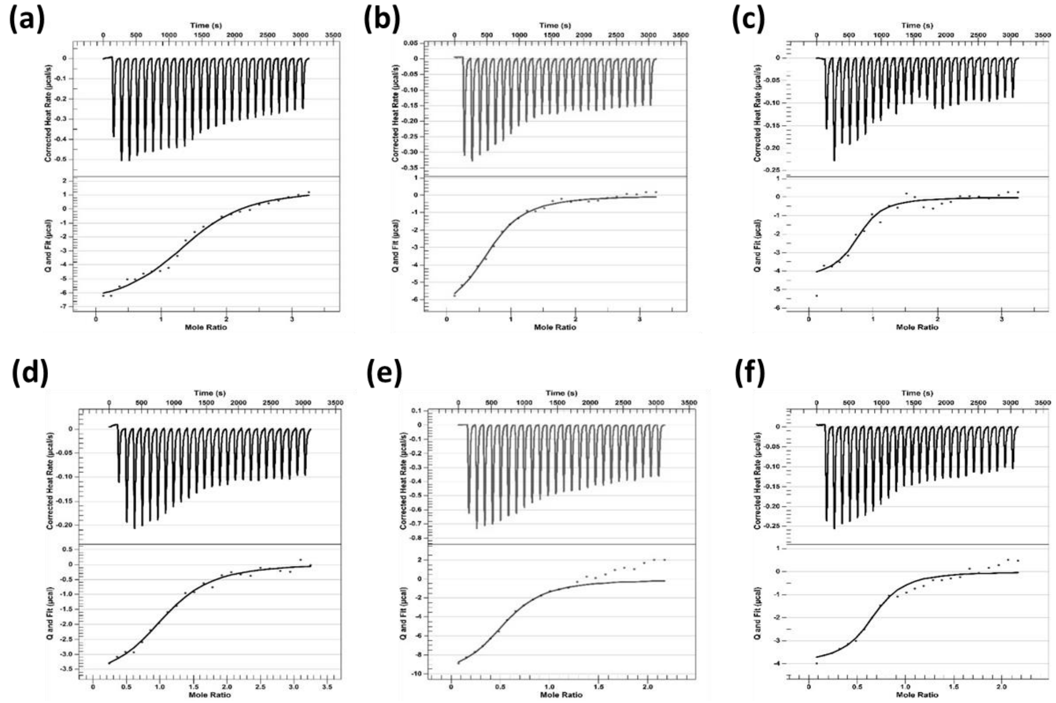

Figure S9. ITC fitting curve of Telomere G1 with **1** (a), **2** (b) and **3** (c), c-*myc* with **1** (d), **2** (e) and **3** (f) under dilute condition. 50 mM KH_2PO_4-K_2HPO_4 buffer (pH 7.0) with 40%(v/v) PEG 200 at 25 °C**.**

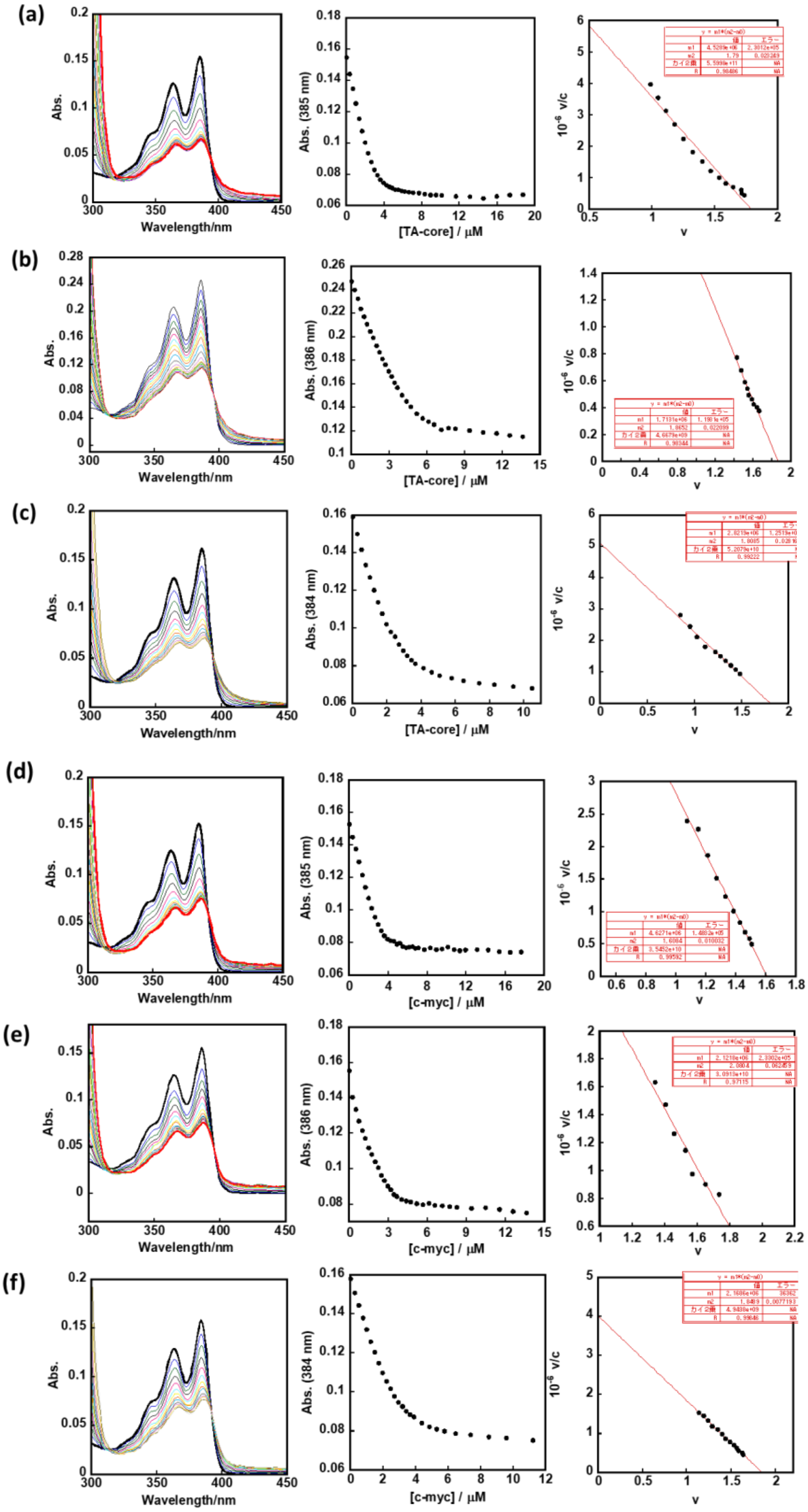

Figure S10. UV-Vis absorbance spectra, titration curve and scatchard plot of Telomere G1 with **1** (a), **2** (b) and **3** (c), c-*myc* with **1** (d), **2** (e) and **3** (f) under dilute condition. 50 mM Tris-HCl buffer (pH 7.4) and 100 mM KCl for telomere G1, 50 mM Tris-HCl buffer (pH 7.4) and 5 mM KCl for c-*myc* at 25 °C.

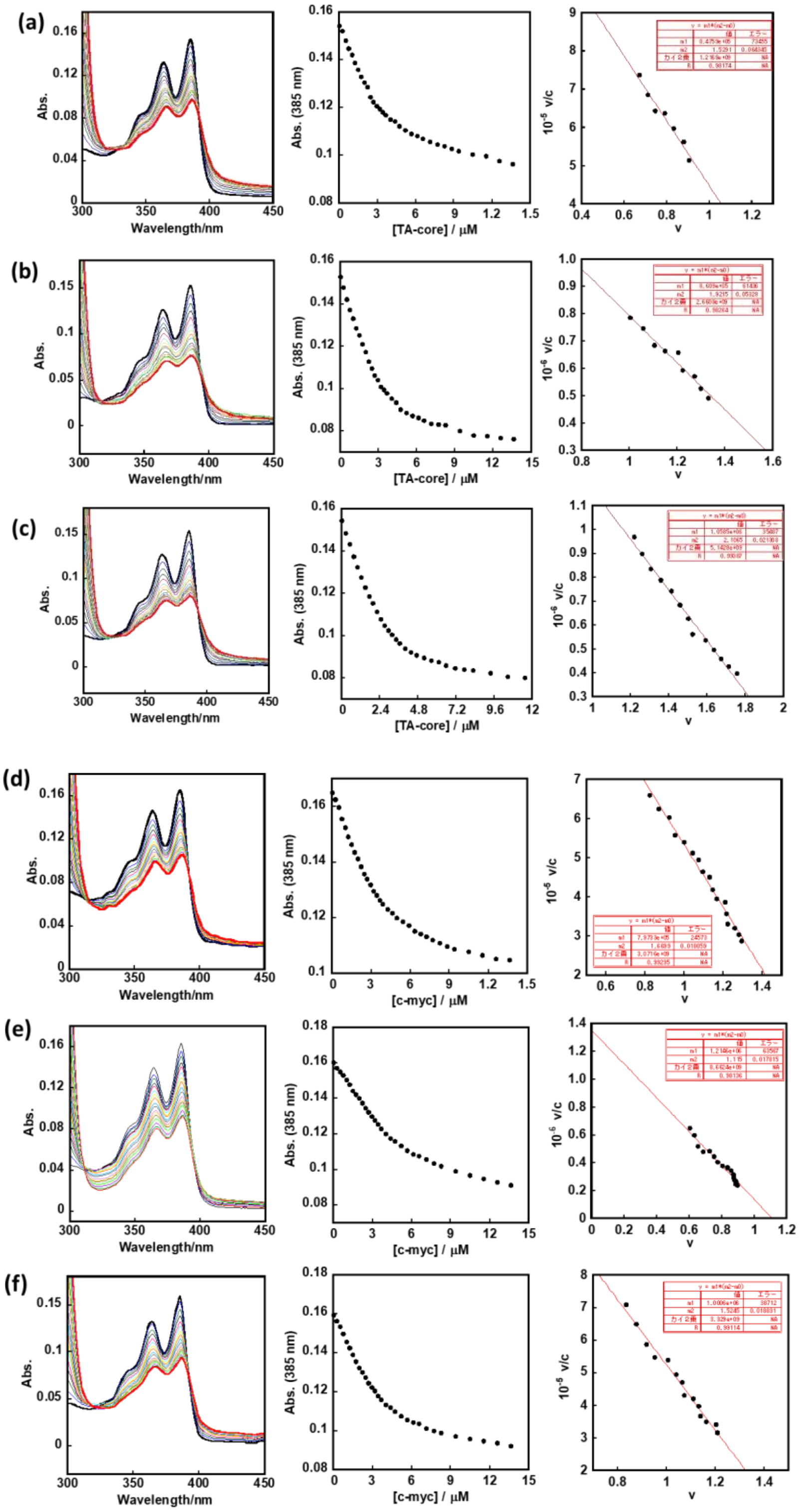

Figure S11. UV-Vis absorbance spectra, titration curve and scatchard plot of Telomere G1 with **1** (a), **2** (b) and **3** (c), c-*myc* with **1** (d), **2** (e) and **3** (f) under molecular crowding condition. 50 mM Tris-HCl buffer (pH 7.4), 100 mM KCl and 40%(v/v) PEG 200 for telomere G1, 50 mM Tris-HCl buffer (pH 7.4), 5 mM KCl and 40%(v/v) PEG 200 for c-*myc* at 25 °C.

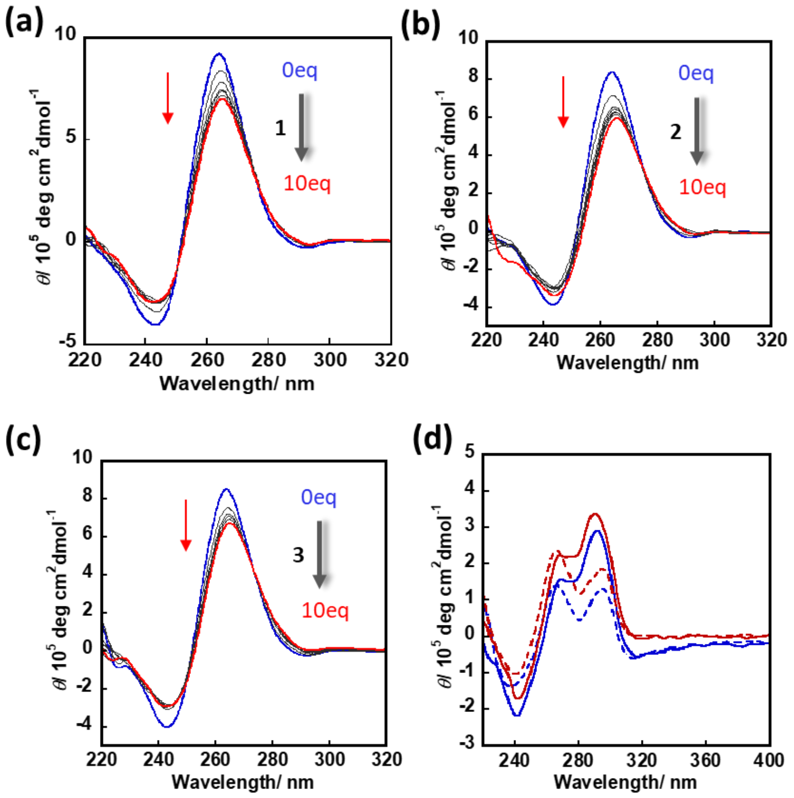

Figure S12. cNDI derivatives enhance the formation of parallel c-*myc* G-quadruplex under cation-deficient molecular crowding condition. Adding **1** (a), **2** (b), **3** (c) to c-*myc* from 0 to 10 equivalents under molecular crowding condition without K^+ (50 mM Tris-HCl buffer (pH 7.4) and 40%(v/v) PEG 200); (d) CD spectra of un-annealed Telomere G1 under molecular crowding condition without K^+, blue dash and red dash line, Telomere G1 only; blue solid and red solid line, Telomere G1 with **1** in 10 eq.

Table S1. CD spectra of Telomere G1 under different condition.

	40%*v*/*v* PEG200	K^+	Annealing	G-quadruplex Structure	CD spectra
**1**	+	+	+	parallel	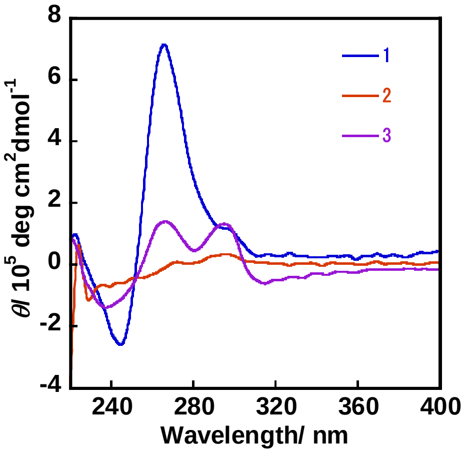
**2**	+	-	+	No signal	
**3**	+	-	-	Parallel/hybrid mixture	
**4**	-	+	+	hybrid	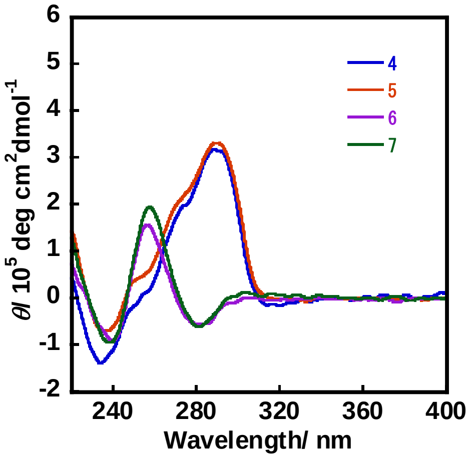
**5**	-	+	-	hybrid	
**6**	-	-	+	ssDNA	
**7**	-	-	-	ssDNA	
